# Miniaturized Stretchable and High-Rate Linear Supercapacitors

**DOI:** 10.1186/s11671-017-2215-5

**Published:** 2017-07-06

**Authors:** Wenjun Zhu, Yang Zhang, Xiaoshuang Zhou, Jiang Xu, Zunfeng Liu, Ningyi Yuan, Jianning Ding

**Affiliations:** 1 Jiangsu Collaborative Innovation Center of Photovoltaic Science and Engineering, Changzhou University, Changzhou, 213164 China; 2 Micro/Nano Science and Technology Center, Jiangsu University, Zhenjiang, 212013 China; 3 State Key Laboratory of Medicinal Chemical Biology, Nankai University, Tianjin, 300071 China

**Keywords:** Wearable electronic, Stretchable linear supercapacitor, High rate

## Abstract

Linear stretchable supercapacitors have attracted much attention because they are well suited to applications in the rapidly expanding field of wearable electronics. However, poor conductivity of the electrode material, which limits the transfer of electrons in the axial direction of the linear supercapacitors, leads to a serious loss of capacity at high rates. To solve this problem, we use gold nanoparticles to decorate aligned multiwall carbon nanotube to fabricate stretchable linear electrodes. Furthermore, we have developed fine stretchable linear supercapacitors, which exhibited an extremely high elasticity up to 400% strain with a high capacitance of about 8.7 F g^−1^ at the discharge current of 1 A g^−1^.

**Electronic supplementary material:**

The online version of this article (doi:10.1186/s11671-017-2215-5) contains supplementary material, which is available to authorized users.

## Background

With increasing development of miniaturized electronic devices, the research on integrated power supplies become more urgent to meet the demanding applications, including micro-robots, smart bracelets, and strain sensors [1–3]. Miniaturized supercapacitors with high-rate performance are a promising candidate for powering these future devices [4, 5]. Moreover, linear supercapacitors have attracted much attention because their flexibility is well suited for wearable electronics [6, 7]. However, these fiber-shaped energy devices have to experience a dramatically stretching process in practical wearable applications. Therefore, it is necessary to evaluate their properties when they are dynamically stretched. Carbon nanotubes are the more suitable for the electrode materials of linear supercapacitors [8–10]. However, energy density of supercapacitors is not high, which hindered the further development of the linear supercapacitors in the field of wearable devices. In order to improve the energy density of supercapacitors, it is common to use pseudocapacitive material to modify electrodes, such as conductive polymers (e.g., PANI, PPy) or metal oxides (e.g., MnO_2_) [9, 11–14]. However, linear supercapacitors suffer a severe loss of capacity at high rates due to the trade-off of axial electron transport. Optimizing axial conductivity of electrodes is a key to circumvent this trade-off. Compared with the flexible linear supercapacitors, the stretchable linear supercapacitors have much poorer rate performances and they are usually tested at low scan rates (0.01–0.1 V s^−1^) [10, 11, 13]. Therefore, it is a key to improve the rate performance of the stretchable supercapacitors.

In this study, we fabricate a kind of stretchable linear supercapacitor based on aligned carbon nanotube (CNT) electrodes. To improve the conductivity of linear electrodes, we employed gold nanoparticles (AuNPs) to modify CNTs. The developed stretchable linear supercapacitor exhibited an extremely high elasticity up to 400% strain with a high capacitance of about 8.7 F g^−1^ at the discharge current of 1 A g^−1^.

## Methods

### Fabrication of PANI@Au@CNT Sheet

An aligned CNT sheet was drawn from an aligned CNT array (with heights of 350 μm and outer diameters of 9 nm) and simultaneously placed on a rectangular rack. The sheet resistance of a single CNT layer was about ~700–1000 Ω/cm, depending upon the areal density of the CNT sheet (which is a function of the forest height) [15]. A thermal evaporation system (MINI-SPECTROS, Kurt J. Lesker, U S A) was used to deposit AuNPs on CNTs to prepare Au_*x*_@CNT sheet (*x* represents the deposition time of Au). To fabricate PANI@Au_*x*_@CNT sheet, polyaniline (PANI) was electrodeposited onto the aligned Au_*x*_@CNT sheets by immersing the Au_*x*_@CNT sheet into an aqueous solution of aniline (0.1 M) and H_2_SO_4_ (1 M) at 0.75 V.

### Preparation of Fine Stretchable Supercapacitors

**Fig. 1:**
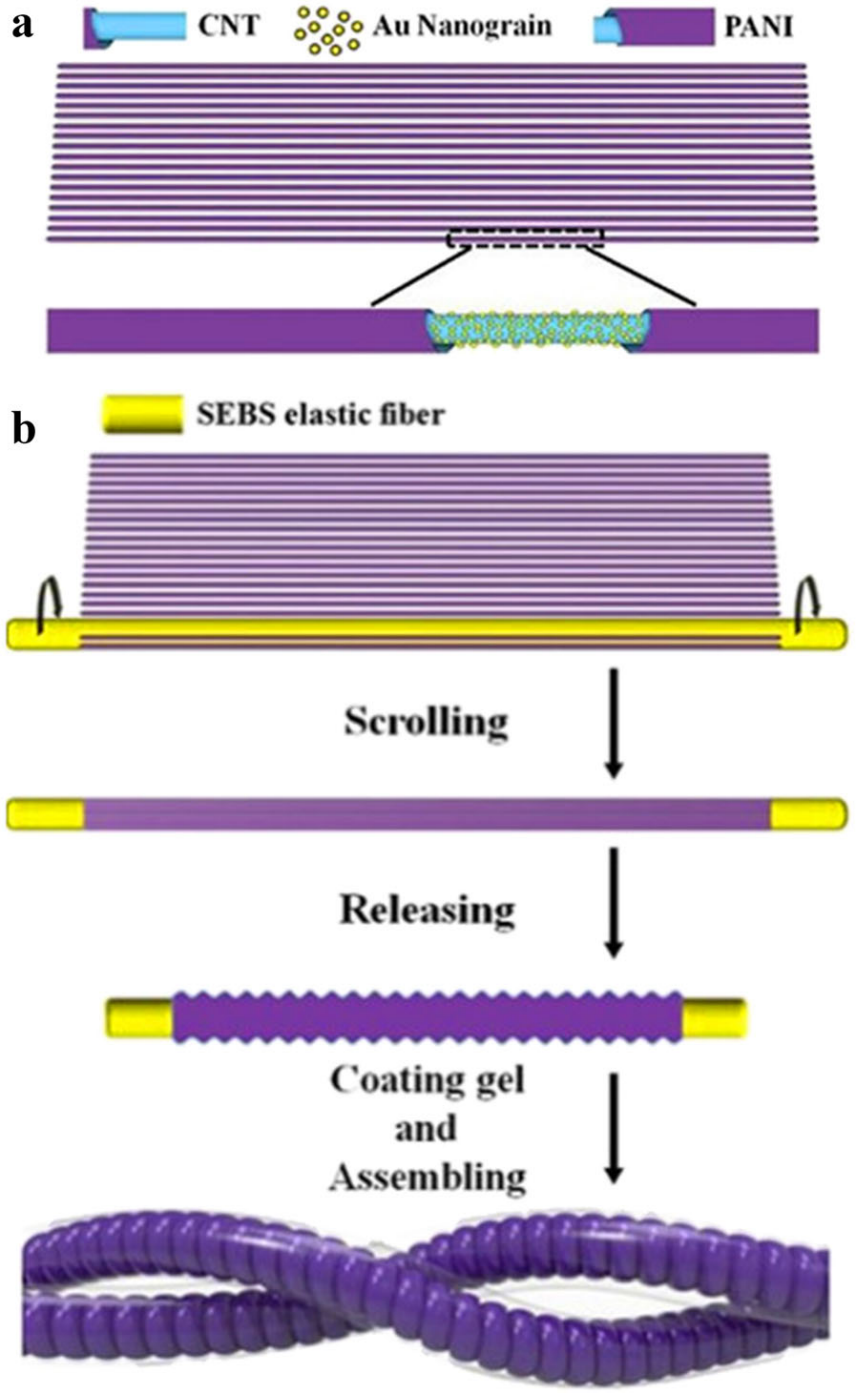
**a**, **b** Fabrication process of the stretchable linear supercapacitors

The fabrication process of stretchable supercapacitors is illustrated in Fig. 1. First, a fine elastic fiber with homogeneous diameter (~200 μm) was prepared using our reported method [16]. Then, the elastic wire was stretched to 400% of its original length and tied between two motor shafts. The motors rotated the fine stretched fiber at a uniform speed to attach the PANI@Au_*x*_@CNT layers onto the rubber fiber. It was important that the CNT direction coincided with the axial direction of the elastic fiber. After wrapping, the strain on the stretched rubber fiber was slowly released to form the non-stretched PANI@Au_*x*_@CNT@fiber.

Finally, H_3_PO_4_/PVA gel electrolyte was prepared and dripped on the surface of PANI@Au@CNT@fiber. After drying for 6 h, the supercapacitor was assembled by twisting two gel-coated electrodes together and then drying for 12 h.

### Characterization

The morphology of the samples was detected by high-resolution field-emission scanning electron microscopy (FE-SEM, Hitachi S4800). The mass content of Au and C in Au@CNT was detected by an energy dispersive spectrometer (EDS) equipped on Hitachi S4800. The electrochemical performance of the stretchable supercapacitors was investigated by electrochemical cyclic voltammetry (CV), and galvanostatic charge-discharge (GCD) using CHI 660E electrochemical workstation. For the three-electrode system, an Au@CNT sheet or a PANI@Au@CNT sheet was used as a working electrode, with a potassium chloride-saturated Ag/AgCl reference electrode and a platinum wire counter electrode. All three-electrode measurements were performed in 1 M H_2_SO_4_ aqueous electrolyte.

## Results and Discussions

**Fig. 2:**
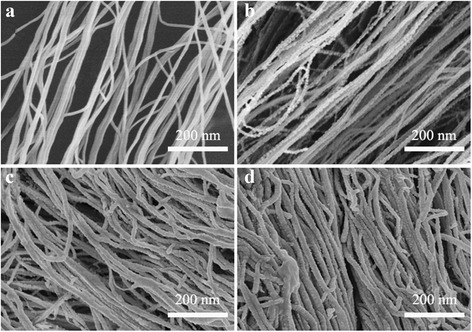
High-resolution SEM images of **a** bare CNT, **b** Au_5_@CNT, **c** Au_15_@CNT, and **d** Au_20_@CNT

**Fig. 3:**
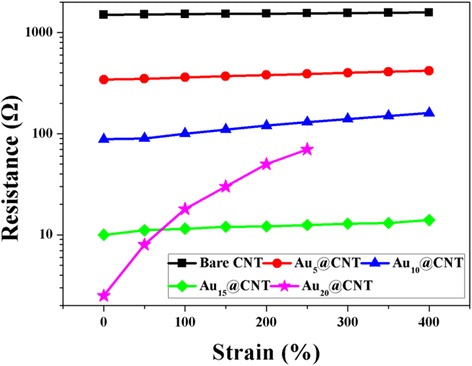
The strain dependence of electrical resistance for CNT@fiber and Au_*x*_@CNT@fiber

Figure 2 shows SEM images of Au@CNT sheets with different deposition time of 5–20 min. It can be seen that bare aligned CNT sheet owns a smooth surface. The results of depositing AuNPs for 5, 15, and 20 min are shown in Figs. 2b–d, respectively. The mass contents of Au and C in Au_*x*_@CNT sheet are shown in Table 1. The results show that the amount of AuNPs distributed on CNTs increased with increasing deposition time. These nanoparticles evenly anchored on the surface of CNT. When the depositing time is 5 min, these nanoparticles are generally independent with each other. With an increase of Au, these nanoparticles connected with each other and covered on the surface of CNT. The amount of AuNPs distributed on CNTs increased with the deposition time increasing, and resulting in a continued decrease on resistance of CNT sheets (Fig. 3). Figure 3 shows the dependence of electrical resistance on applied strain for Au@CNT@fibers. Au_20_@CNT@fiber showed a low electrical resistance but reduced stretching ability. When the applied strain reached 250%, the electrical resistance increased more than 100%. In comparison, the applied strains of 0–400% caused no significant change in resistance of Au_15_@CNT@fiber.

**Table 1:** Mass content of Au and C in Au_*x*_@CNT sheet (*x* = 5, 10, 15, 20)

	Au_5_@CNT	Au_10_@CNT	Au_15_@CNT	Au_20_@CNT
Au (wt. %)	93.2	96.1	97.8	98.7
C (wt. %)	6.8	3.9	2.2	1.3

**Fig. 4:**
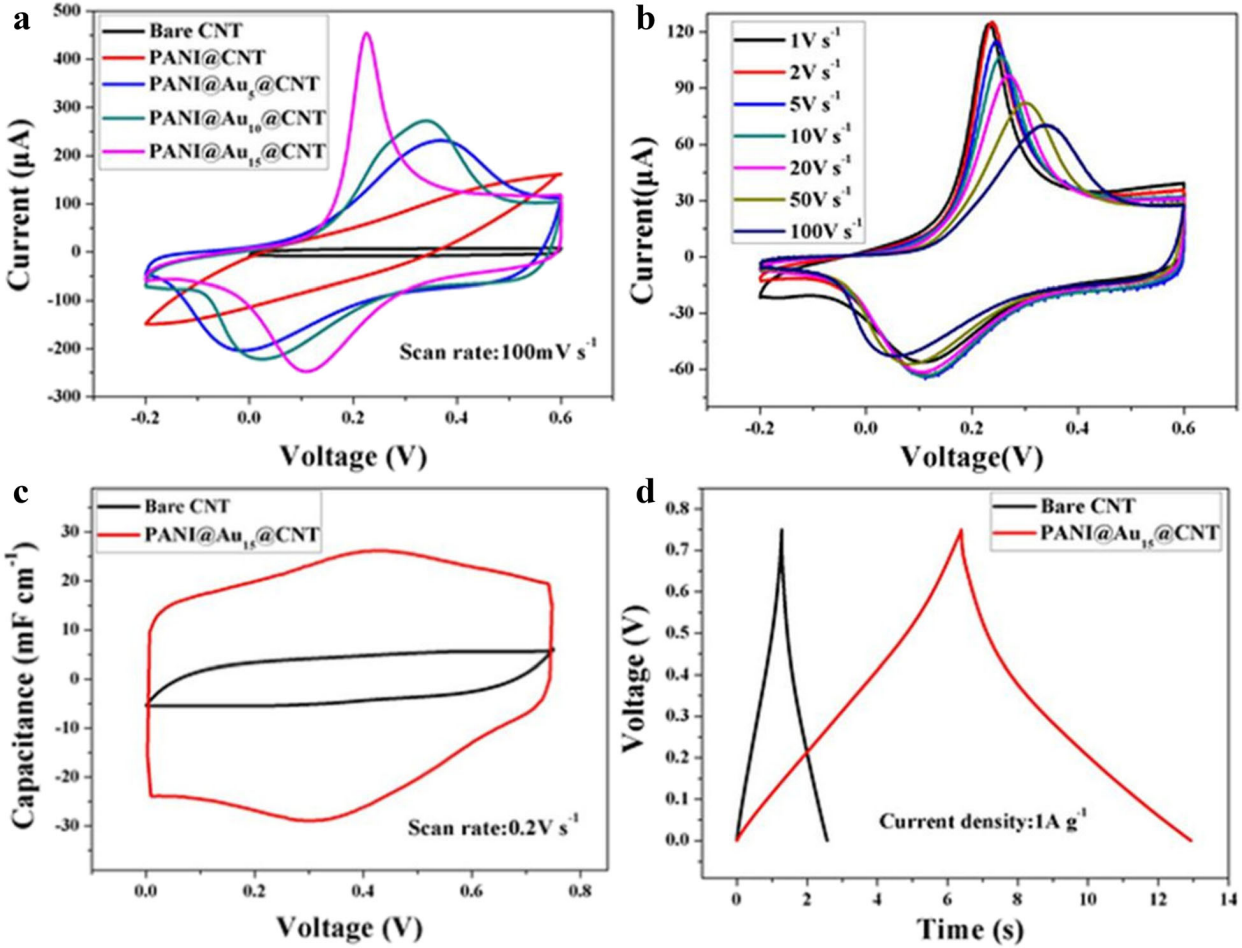
**a** CV curves of bare CNT and PANI@Au_*x*_@CNT electrode materials at a scan rate of 100 mV s^−1^; **b** CV curves of PANI@Au_15_@CNT at a scan rate of 1–100 V s^−1^; **c** CV curves of supercapacitors based on the bare CNT and PANI@Au_15_@CNT at a scan rate of 200 mV s^−1^; **d** GCD curves of supercapacitors based on the bare CNT and PANI@Au_15_@CNT electrodes at a current density of 1 A g^−1^. The CV curves obtained from different scan rates are normalized to 1 V s^−1^ in **b**

Figure 4a shows three-electrode measurements of both the bare CNT sheet and PANI@Au_*x*_@CNT sheet (*x* = 0, 5, 10, 15) at a scan rate of 100 mV s^−1^. The high conductivity of Au_15_@CNT sheet facilitates the rapid transport of electrons, thus improving the rate performance of PANI@Au_15_@CNT sheet greatly. Therefore, in the following work, PANI@Au_15_@CNT sheet was selected as electrode material for further CV testing with the scan rate from 1 to 100 V s^−1^. For comparison, the normalized capacitance as a function of the scan rate for CNT, CNT@Au_15_, CNT@PANI, and PANI@Au_15_@CNT are shown in Additional file 1: Figure S1(a). Figure 4b shows that the redox potentials of PANI remain constant with an increasing scan rate from 1 to 100 V s^−1^; it indicates that PANI here undergoes a rapid redox reaction, thus enhancing the power characteristics of the electrode material [17, 18].

Figure 4c shows CV curves of wire-like symmetrical supercapacitors of CNT@fiber and PANI@Au_15_@CNT@fiber, respectively. A distinct difference between these two supercapacitors indicates a great improvement of capacitive behavior of PANI@Au_15_@CNT@fiber. Figure 4d shows GCD curves of these two symmetric supercapacitors. The symmetric triangular shape indicates that both two supercpacitors own a good supercapacitive performance. The specific capacitance of the CNT-based supercapacitor was about 1.6 F g^−1^ at the current density of 1 A g^−1^, for PANI@Au_15_@CNT-wrapped electrode, this value was about 8.7 F g^−1^. In order to ensure the accuracy of capacitance of the electrode materials, we weigh the electrode before and after deposition of PANI. The mass content of PANI is about 46 mg g^−1^ and the capacitance of PANI is about 360.8 F g^−1^.

**Fig. 5:**
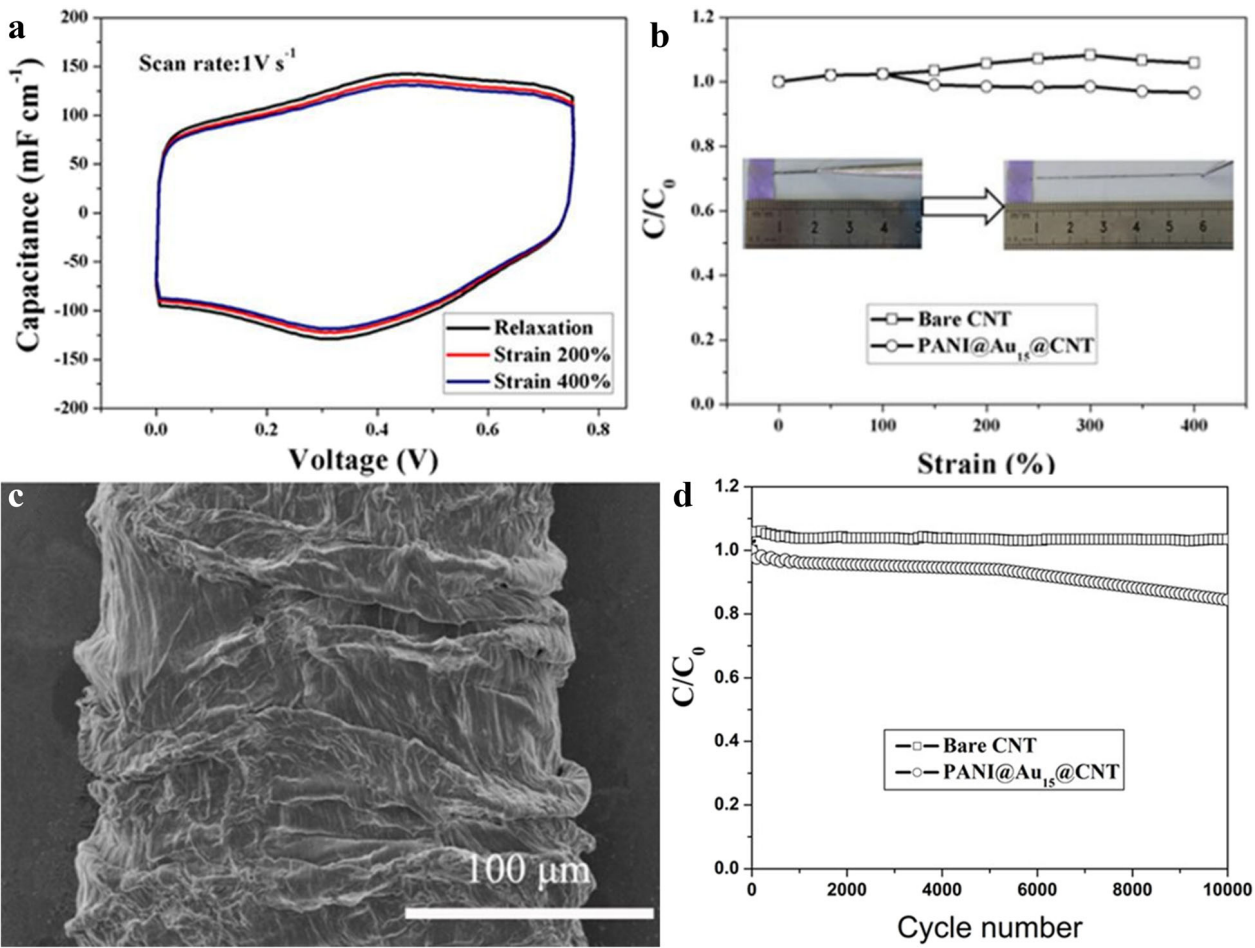
**a** CV curves measured at different states. **b** The normalized capacitance as a function of the tensile strain. **c** SEM image of stretchable electrode at release state. **d** The capacitance for the device based on the bare CNT and PANI@Au_15_@CNT electrodes

Further, the supercapacitive performance of the PANI@Au_15_@CNT-based supercapacitor was measured under different strain rates. As shown in Fig. 5a, similar CV curves indicate that supercapacitive performance of the PANI@Au_15_@CNT-based supercapacitor was not affected greatly under strain state, even when the strain rate increased to 400%. Figure 5b shows the strain-normalized capacitance as a function of the tensile strain. It can be seen that the capacitance for the supercapacitor based on the PANI@Au_15_@CNT@fiber electrodes had no obvious change, while the device based on the CNT@fiber electrodes increased by 10% as the tensile strain increased from 0 to 400%, this may be caused by the strain-induced enhancement in the contact between the two twisted electrodes upon stretching [19]. The good elasticity is ascribed to the buckled structure of PANI@Au@CNT@fiber. For comparison, the normalized capacitance of CNT@Au and CNT@PANI as a function of tensile strain are shown in Additional file 1: Figure S1(b). Figure 5c indicates a buckled structure of a PANI@Au@CNT@fiber in a relaxed state. Figure 5d shows the capacitance change after cycling. For the bare CNT electrodes, nearly no decrease can be found after 10,000 cycles, whereas for PANI@Au15@CNT electrode, the capacitance decreased about 10% after 10,000 cycles. The performance of the extremely stretchable wire-shaped supercapacitors developed here exceeded that of previously reported state-of-the-art stretchable electronic systems, regarding both elasticity and rate performance [13, 14, 20].

## Conclusions

In this work, a fine stretchable linear supercapacitor based on PANI@Au@CNT@fiber electrodes was fabricated. The fabricated supercapacitor can undergo strain of up to 400%. The supercapacitor based on PANI@Au_15_@CNT@fiber electrodes was approximately 8.7 F g^−1^ at the discharge current of 1 A g^−1^. The stretchable supercapacitors also showed a long-term stretching stability after 1000 stretching cycles and long life after 10,000 charge-discharge cycles.

## Additional File

Additional file 1: Supporting information. (DOCX 21 kb)
