# Supplementary material for: Miniaturized Stretchable and High-Rate Linear Supercapacitors
Source: Nanoscale Res Lett. 2017 Jul 6;12:448. doi: 10.1186/s11671-017-2215-5 (PMC5500600; doi:10.1186/s11671-017-2215-5)
Supplement: Additional file 1: — Supporting information. (DOCX 21 kb) [file 11671_2017_2215_MOESM1_ESM.docx]

**Supporting information**

| Electrode materials | Elasticity | Capacitance | Refs. |
| --- | --- | --- | --- |
| PANI@Au_15_@CNT sheet | 400% | 8.7 F g^-1^ | This work |
| CNT | 100% | 19 F g^-1^ | [[1](#_ENREF_1)] |
| SWCNT/PANI hybrid electrodes | 140% | 106 F g^-1^ | [[2](#_ENREF_2)] |
| CNT/PEDOT-PSS composites | 350% | 8.0/30.8 F g^-1^ | [[3](#_ENREF_3)] |
|  |  |  |  |

1. Yang Z, Deng J, Chen X, Ren J, Peng H: **A highly stretchable, fiber-shaped supercapacitor**. *Angew. Chem.* 2013, **52:** 13453-13457.

2. Zhang N, Luan P, Zhou W, Zhang Q, Cai L, Zhang X, Zhou W, Fan Q, Yang F, Zhao D, Wang Y, Xie S: **Highly stretchable pseudocapacitors based on buckled reticulate hybrid electrodes**. *Nano Research* 2014, **7:** 1680-1690.

3. Chen T, Hao R, Peng H, Dai L: **High-performance, stretchable, wire-shaped supercapacitors**. *Angewandte Chemie* 2015, **54:** 618-622.
